# Perceptions of integrated rehabilitation service delivery in a metropolitan district

**DOI:** 10.4102/phcfm.v16i1.4069

**Published:** 2024-01-29

**Authors:** Lebogang J. Maseko, Fasloen Adams, Hellen Myezwa

**Affiliations:** 1 Department of Occupational Therapy, School of Therapeutic Sciences, Faculty of Health Sciences, University of the Witwatersrand, Johannesburg, South Africa; 2 Department of Health and Rehabilitation Sciences, Division of Occupational Therapy, Faculty of Medicine and Health Sciences, Stellenbosch University, Stellenbosch, South Africa; 3 Department of Physiotherapy, School of Therapeutic Sciences, Faculty of Health Sciences, University of the Witwatersrand, Johannesburg, South Africa

**Keywords:** occupational therapy, physiotherapy, speech therapy, audiology, universal health coverage, service delivery, disability

## Abstract

**Background:**

There is a recognised need for rehabilitation services at primary health care (PHC) level. In addition, there are clear policies (international and national) and guidelines for use by healthcare planners in South Africa to implement rehabilitation services. Although rehabilitation services are provided on the primary platform, its operationalisation has not been in an integrated manner. Clarity on the level of integration within existing PHC rehabilitation service delivery is required for its inclusion in a reengineered PHC.

**Aim:**

The study explored the extent to which rehabilitation services are integrated into PHC service delivery based on the expressed reality of rehabilitation professionals.

**Setting:**

The Johannesburg Metropolitan District of Gauteng, South Africa.

**Methods:**

In-depth interviews with 12 PHC rehabilitation professionals were completed to elicit their experiences with PHC rehabilitation services.

**Results:**

The theme *the current state of rehabilitation services – ‘this is the reality; you need to do what you need to do’* along with its two subcategories, was generated from this study. The theme describes the expressed reality of suboptimal, underdeveloped and poorly integrated rehabilitation services within the Johannesburg Metropolitan District. Rehabilitation service providers have adapted service delivery by including isolated components of rehabilitation integration models, but this has not yielded an integrated service.

**Conclusion:**

Rehabilitation services although recognised as a crucial service in PHC must be critically analysed and adapted to develop integrated service delivery models. There should be a shift from selected coping mechanisms to targeted, integrated services.

**Contribution:**

The study describes PHC rehabilitation services and explores best practice models for integrated service planning and delivery.

## Introduction

As the global health community strives for universal health coverage (UHC) and health equity in line with the 2030 Agenda for Sustainable Development,^1^ there is renewed interest in primary health care (PHC) informed by evidence from research.^2^ Providing a continuum of healthcare services at the community level improves mortality (maternal, newborn and neonatal), life expectancy, mental health outcomes and reduces hospitalisations.^3^ The principles of PHC include equity, access, affordability and appropriateness of health services, empowerment of people and sustainability of service provision.^4,5^ The strength of PHC lies in its adaptability to local needs where communities are the unit of intervention through a comprehensive intersectoral approach,^4^ making it particularly relevant for integrating healthcare services.

Although existing international and national policies and guidelines recognise the need for multidisciplinary PHC, which includes rehabilitation services, they fail to address the extent to which rehabilitation services should be integrated, organised and delivered,^6,7^ There is a misperception that rehabilitation is a luxury and not essential care.^8^ According to the World Health Organization (WHO) global disability action plan, rehabilitation must be addressed as a necessary service rather than a luxury supplement to promote the integration of rehabilitation into PHC.^9^ However, services delivered within a vertical medical model of care based on diagnosis act as a barrier to the effective integration of all services including rehabilitation at a PHC level.^10^ A biopsychosocial approach considering context-related problems as well as the functional outcomes in relation to health^11^ that places the needs of the people at the centre of the system^12^ is required to support integration of services.

The need to scale up rehabilitation services at the PHC level to achieve UHC for persons with disabilities or at risk of disability is articulated clearly in the second objective of the WHO Global Disability Action Plan 2014–2021 ‘Better health for all people’.^9^ In South Africa, the public health system is largely inadequate in the provision and integration of PHC rehabilitation services.^13^ Inadequate service provision in South Africa results in a higher rate of unmet health and community integration needs,^14^ confounded by geographical,^15^ infrastructural,^16^ attitudinal^17^ and financial barriers^18^ that continue to affect access to healthcare. In addition, participation in daily life as an important outcome to facilitate independence, social inclusion and quality of life for those with functional limitations is lacking in service provision.

Conceptual ambiguity of integrated rehabilitation might be another hindrance to rehabilitation professionals’ holistic understanding of the suitability of the service at the PHC level.^19^ Consequently, rehabilitation professionals rely on their own experiences and conceptual understanding of the situation to guide their practice.^19^ To explore and clarify integration of rehabilitation in PHC, systems and services should be considered over three levels, namely macro, meso and micro levels.^12^ Integration takes place at the macro level and refers to the alignment of policies and laws within a system, including legal frameworks or national instruments, such as the Framework and Strategy for Disability and Rehabilitation Services in South Africa.^7^ Integration at the meso level occurs both professionally and organisationally with partnerships or teams coordinating services across various disciplines, both within and between organisations.^12^ Integration at this level implies bringing together health with other agencies such as housing, social services and transportation.^20^ The micro level refers to clinical integration in providing care to individual patients and communities.^12^ The need for comprehensive care is greatest at the micro level where information, decision-making and service delivery responsibilities are shared by all health professionals^21^ specific to the person’s individual requirements. Micro-level integration supported by connectivity is achieved through intersectoral collaboration and a shared mission, vision, values and culture between organisations, professional groups and individuals.^12^

The advantage of integrated services in PHC including rehabilitation is the effective use of the combined expertise of the team rather than healthcare workers practising individually.^20^

Table 1 provides a summary of the six models of practice for providing rehabilitation services in an integrated approach in PHC as described by McColl et al.^22^ It describes the key features of the model, its advantages and disadvantages.^22^ While the literature was derived mainly from the global north, one can argue that the models are or could be implemented in varying degrees in global south countries too.

**TABLE 1:** The six models for integrated rehabilitation services delivery in primary health care.

Model	Key features	Advantages	Disadvantages
Clinic model	Family physicians or GPs, PHC nurses, and rehabilitation professionals are co-located (in the same physical place), resulting in a geographically defined team.	Efficient from the professional’s perspective and affords the opportunity for joint appointments if necessary^22^	Removes patients from their social context, limiting the biopsychosocial approach
Outreach model	Emanate from an institutional base and concentrate on providing professional services to people who could not access them in their usual institutional location.	Often target remote or resource-poor locations and attempts to simulate service offerings without the infrastructure provided by the facility by using mobile teams and satellite units	If the outreach teams are not integrated into the community they serve, they may be perceived as visiting services
Self-management model	Systematic provision of education and support by health care staff to increase patients’ skill and confidence in managing their health problems	Involves the service users’ in managing their own health and is compatible with the rehabilitation philosophy of optimising independence	While compatible with the rehabilitation philosophy of optimising independence,^22^ self-management requires health literacy, which is low in South Africa and other low- and middle-income countries^24^
Case management model	A case manager, on the basis of a referral and intake assessment, marshals and coordinates the necessary services, including family medicine and rehabilitation services, either in the patient’s home or other community location	Coordinates services, including rehabilitation services at the PHC facility, the patient’s home or other community locations, such as residential homes	Effective case management relies on an understanding of rehabilitation, its value and place in patient care and cooperation from other professionals involved in the patients’ care
Community-based rehabilitation (CBR) model	Based on a community development philosophy, the CBR model uses an interdisciplinary and transdisciplinary collective approach. The role of rehabilitation professionals in using this model is to advocate with people with chronic diseases and disabilities to mobilise community resources and address community needs.^25^	Typically results in broader, more far-reaching effects than could be achieved on a one-to-one basis. The development of relationships between the rehabilitation team and other members of the primary care team is a key issue.^23^	Typically, community development is a process that requires a commitment of time and energy over a sustained period. The absence of collaborative relationships threatens not only the sustainability of CBR but the effective integration of rehabilitation services.
Shared care model	Occurs when specialist professionals pair with a generalist or novice practitioner to collaborate on patient management.	Inclusion of rehabilitation services in an integrated shared model of service delivery has potential benefits for interprofessional teamwork.	No analogous model found of specialist-generalist collaboration between rehabilitation personnel in the primary care and tertiary care settings. Thus, the efficiency of a shared care model requires further examination.

*Source*: McColl MA, Shortt S, Godwin M, Smith K, Rowe K, O’Brien P, Donnelly C. Models for integrating rehabilitation and primary care: A scoping study. Arch Phys Med Rehabil. 2009;90(9):1523–1531. https://doi.org/10.1016/j.apmr.2009.03.017.

PHC, primary health care; GP, general physician.

In South Africa, the lack of rehabilitation personnel and the employment of newly qualified rehabilitation professionals, completing a year of community service, in clinic and outreach services results in high staff turnover and little opportunity for long-term skill development. Services are often contextually inappropriate, unsuited to community needs and lack experienced staff who have the skills to integrate services into the community.

Interprofessional trust is central to the success of collaborative relationships within the medical workforce^26^ and requires knowledge of the role, competencies, strengths and limitations of other health workers in the team. As outlined in many of the PHC rehabilitation integration models, interprofessional trust is essential for multidisciplinary teamwork and takes time, professional maturity and patience to develop. Contrasting professional values could potentially affect the development of relationships between health professionals and negatively influence the delivery of integrated rehabilitation services in PHC. While rehabilitation is team-orientated in theory,^18^ not all healthcare professionals apply it in practice, therefore, limiting interprofessional referral and collaborative partnerships that could benefit patient care.^26^

In South Africa, national policies and guidelines^6,27,28^ prescribe the integration of rehabilitation services in PHC but are silent on how to achieve integration. At present, this gap in policies and the absence of research that reports on the extent of integration of rehabilitation services has contributed to the lack of knowledge in rehabilitation service integration in South Africa. To be successful within PHC, rehabilitation must be collaborative, comprehensive, efficient, cost effective and justified by evidence.^29^ Using integration as a core PHC principle is a means to investigate the inclusion of accessible public health for all to achieve the Sustainable Development Goals (SDGs) 2030.^30^ The WHO *Rehabilitation in Health Systems: Guide for Action (2019)*^8^ emphasises the need for research to ensure the provision of rehabilitation services integrated into health governance, services planning, financing and health workforce initiatives. Identifying and clarifying the needs of the clinical service and setting priorities for action are suggested as the first steps to strengthen rehabilitation and enact policies for integration into PHC. Rehabilitation services are currently delivered at the PHC facilities in the Johannesburg Metropolitan District. The extent to which rehabilitation is recognised by policy makers and other PHC service providers as an essential component of integrated PHC service delivery is unknown. The study aimed to explore the extent of integration of rehabilitation services in healthcare provision at the PHC level according to the expressed realities of rehabilitation professionals in a specific setting, the Johannesburg Metropolitan District.

## Research methods and design

### Study design

To explore the realities of the integration of rehabilitation services in PHC from the perspective of the rehabilitation service providers, a descriptive exploratory qualitative approach was followed.^31^

### Study setting

The study was conducted in the Johannesburg Metropolitan District, which comprises 125 primary health care clinics and community health centers (CHCs) across seven regions.^32^ The district serves a population of 5.5 million, with a significant portion falling below the upper limit of the poverty datum line.^32^ The 81 clinics including 11 CHCs are provincially funded and staffed by the Gauteng Health Department. Nine of these provincial facilities provide rehabilitation services.^33^ Community health centers provide PHC services, 24-h maternity services, accident and emergency services and beds where healthcare users can be observed for a maximum of 48 h. By definition, clinics provide a range of PHC services and operate an average of eight or more hours a day based on the needs of the community being served.^34^

### Study population and sampling strategy

Rehabilitation professionals, including managers, employed in the various PHC facilities in the Johannesburg Metropolitan District, were included in the study. Purposive sampling with stratification was used to recruit a heterogeneous sample of experienced rehabilitation professionals.^35^ The sample included participants from the occupational therapy, physiotherapy, speech therapy and audiology professions with work experience that they could draw on. Participants were required to have a minimum of 2 years’ working experience in PHC at the clinic level to ensure that participants had sufficient rehabilitation service provision experience, allowing them to comment on these services. Rehabilitation managers needed at least 5 years’ experience at a managerial level. It was postulated that management needed an in-depth understanding of not just the micro context but also the macro and meso contexts. Therefore, it would take longer for this knowledge to develop in an in-depth way and thus the use of a five-year experience criterion for managers. Participants were contacted directly through the rehabilitation coordinators at the PHC facilities and were invited to participate in the study in their personal capacity.

### Data collection

In-depth interviews were conducted with the selected participants to gather data. The interviews were carried out at the various clinics as well as a central venue convenient for the participants, when privacy at the clinics was not adequate. The participants were asked to share their views on the current state of rehabilitation services in the district. An interview guide was developed to serve as prompts for the discussion points. The interviews were conducted in English, a common language among the participants.

### Data analysis

The audio-recorded data from the interviews were transcribed verbatim. An inductive approach was used for data analysis, employing NVivo software version 11 (Lumivero). Primary inductive coding of the data was initially conducted based on the respondents’ words. Higher-order coding was then applied to consolidate the primary codes into subcategories.^36^ Thematic development was performed to organise the higher-order codes into categories and themes. Excerpts from the raw data were included to support the overarching themes and ensure that the interpretation remained connected to the participants’ words.^37^

### Ethical considerations

Ethical approval for the study was obtained from the University of the Witwatersrand Committee on Human Research Ethics (Medical) as well as the Johannesburg Health District Research Committee (M190466). Purposive sampling of the rehabilitation service providers aimed to ensure transferability of the findings.^36^ Two coders were involved in the coding process, the researcher and an experienced qualitative researcher. Inter-coder agreement was established using a codebook. The coders developed preliminary codes of the transcripts separately and then compared and agreed on the coding. Prolonged engagement and data saturation were achieved to enhance credibility.^36^ Themes were built around the identified codes using a process of consensus.^38^ In addition, the analysed transcriptions were discussed with an experienced researcher, and the transcripts were availed to an independent researcher not involved in the study who conducted independent analysis and coding to improve the trustworthiness of the findings.^39^ The researcher maintained a reflexive diary to reflect on her own role and minimise biases. Member checking was performed by sending transcribed notes to the participants for verification.^39^

## Results

The majority of the 12 participants were female (*n* = 10), occupational therapists (*n* = 5), in clinical roles (*n* = 6), rather than managerial ones and had between 6 and 10 years of experience (Table 2). The participants worked in 9 of the 81 provincially funded PHC facilities in the Johannesburg Metropolitan District and 8 of these facilities are CHCs and 1 is a clinic. The nine facilities are distributed across the five sub-districts in the Johannesburg Metropolitan District.

**TABLE 2:** Demographic information and characteristics of the participants according to their profession, position, years of experience, sex and highest professional qualification achieved (*n* = 12).

Participant characteristics	Rehabilitation professions			
	OT (*n*= 5)	PT (*n* = 4)	ST&A (*n* = 2)	SLT (*n* = 1)
**Position**				
Clinical	2	2	1	1
Management	1	1	0	0
Both	2	1	1	0
**Years of experience**				
2–5	1	0	2	2
6–10	3	3	1	1
11+	1	1	0	0
**Sex**				
Male	0	2	0	0
Female	5	2	2	1
**Highest qualification**				
Bachelors	4	1	2	2
Masters	1	3	0	0

OT, occupational therapy; PT, physiotherapy; ST&A, speech therapy and audiology; SLT, speech and language therapy.

A theme of *the current state of rehabilitation services – ‘this is the reality; you need to do what you need to do’* was generated from the data on the participants’ perception and experiences of the integration and comprehensiveness of rehabilitation services in PHC in the Johannesburg Metropolitan District. The theme expressed various suboptimal aspects in the delivery of rehabilitation services in the Johannesburg Metropolitan District and shows the creative and adaptable strategies used to mitigate the contextual challenges in meeting service users’ needs.

**FIGURE 1:**
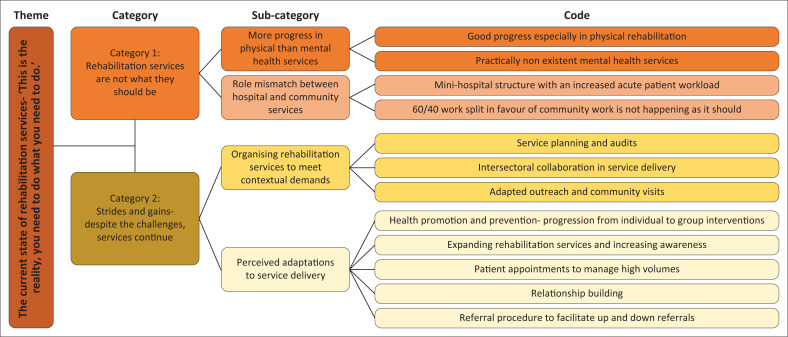
The theme *current state of rehabilitation services – ‘this is the reality; you need to do what you need to do’,* with its corresponding categories, sub-categories, and codes depicting the perceived sub-optimal state of rehabilitation services in PHC in the Johannesburg Metropolitan District.

The participants’ descriptions within this theme expressed the lived reality, perceptions and experiences of the rehabilitation service at the coalface: the state of integration of rehabilitation services into the health service in PHC and the comprehensiveness of the service. The participants expressed having to do their best pragmatically (Figure 1).

### Category 1: Rehabilitation services are not what they should be

This category speaks to the lack of comprehensive and holistic services. According to the participants, rehabilitation services in the Johannesburg Metropolitan District are suboptimal. Services are characterised by differences between physical and mental health services and a role mismatch between hospital and community-based services.

#### Subcategory 1.1: More progress in physical than mental health services

The first subcategory described the discrepancies between the physical and mental healthcare services. The former was described to be more established than the latter in the Johannesburg Metropolitan District:

‘In terms of physical rehabilitation … we have made lots of strides.’ (KI 1, female, OT)

In fact, they felt that mental health services are practically non-existent in the district and that the odd activity that takes place is mainly forced by regulations. For example, were it not for the regulations, the mental health audits performed by the rehabilitation professionals at the mental health non-governmental organisations (NGOs) would not take place.

‘… [*I*]n terms of mental rehabilitation, I think it’s close to non-existent … it’s mainly activities that we are forced by regulations that are taking place.’ (KI 1, female, OT)

Participants also felt that the mental health teams lacked the complete complement of the psychosocial multi- and interdisciplinary team that was present in the physical rehabilitation teams:

‘… [*F*]or the mental health teams, they never had the full team. There is always one, you know, this one not there and that one not there.’ (KI 3, female, ST&A)

#### Subcategory 1.2: Role mismatch: between prescribed community health centre services and current community health centre services

Subcategory 1.2 refers to the participants’ views that rehabilitation services in the CHCs duplicated the medical model of hospitals to the point of operating as mini hospitals and that this is exacerbated by an increased caseload of patients with acute conditions or phases of illness:

‘… [*W*]hat it shouldn’t be is a mini-hospital department … You should not want to duplicate what you do in a hospital … at a clinic …’ (KI 2, female, PT)

The mini-hospital concept includes the manner in which services are delivered, and participants emphasised that they see more acute clients who require curative, institution-based, hospital-type services when the participants believed rehabilitation services on the PHC level should be conducting integrated health promotion and prevention of disability campaigns in collaboration with all stakeholders, among other aspects. The increasing referrals of acute patients from hospitals were attributed to the workload pressures and staff shortages in the secondary and tertiary hospitals. Consequently, the 60/40 work split in favour of community work is not happening as it should, because of the influx of acute patients. Therefore, the rehabilitation professionals felt they are forced to focus more on clinic-based work instead of community outreach as intended:

‘So, 60% is supposed to be out in the community, and 40% supposed to be like in the clinic, outpatients. It actually does not always work out that way because we get a lot of acute referrals.’ (KI 3, female, ST&A)

### Category 2: Strides and gains – Despite the challenges, services continue

The second category highlights the persistent nature of rehabilitation professionals who continue to render services despite the challenges in the Johannesburg Metropolitan District. In this district, rehabilitation professionals perceived themselves as adaptable and creative in ensuring service delivery. Despite the participants’ perception that rehabilitation services are suboptimal, they remain committed to assuring rehabilitation service delivery. Their creativity and adaptability allow them to compensate for resource limitations by organising and executing rehabilitation services in accordance with the contextual demands. They also emphasised their perceptions of specific areas of service delivery that have been modified to assure service delivery.

#### Subcategory 2.1: Organising rehabilitation services to meet contextual demands

Rehabilitation professionals reported that they use monthly multidisciplinary timetables and peer audits of services as strategies to mitigate the demands that the increased patient numbers place on them and ensure that services continue to meet the patients’ needs. These strategies include monthly planning within the rehabilitation team where activities such as outreach, community visits and health campaigns are captured on a shared calendar to manage the patient load and staff distribution:

‘So monthly we do a timetable, so we have a plan … of when we are in clinic, when we are out of clinic … on outreach, then … our managers audit those.’ (KI 4, female, OT)

There is also reciprocal peer auditing of aspects such as patient reports and intersectoral collaboration with the Department of Education, for example, in health promotion and prevention campaigns. However, it is not possible for rehabilitation professionals to conduct community visits for at least 60% of their work time as stipulated in their job description. Consequently, outreach and community visits have decreased in frequency:

‘We do it [the audit] as a department … the OT will … grab a couple of the physio’s files … audit ourselves, get our scores, … hand them over to our supervisor.’ (KI 8, female OT)

‘… Department of Education started coming in … who fulfils what roles … a meeting was held with the Department of Education … & Health to … come to this agreement.’ (KI 9, female, OT)

Health campaigns are organised on a much larger scale to reach the target population, and they recognised that close collaboration with various governmental departments, such as social development, extends their reach:

‘So, we run a lot of campaigns … So, we’ll do a three day workshop … getting them to take over a bit of our services … there’s more carryover because those community workers will see the patients more often.’ (KI 10, female, OT)

The expressed mitigation strategies are used as planning and organisation tools to ensure that health promotion and prevention rehabilitation services are delivered despite the constraints of the current contextual challenges.

#### Subcategory 2.2: Perceived adaptations to service delivery

This subcategory describes the activities that the participants felt they have adapted to facilitate the efficient delivery of rehabilitation services in PHC facilities. Included in this is a method to assign patients to receive either individual or group therapy to ensure that services are person centred. Once a patient has received one-on-one therapy for a time period, they are progressed to group therapy where patient-directed models of service delivery such as peer learning and support are used:

‘… [*T*]he patients get seen for a specific number of individual treatments … After they’ve been seen for those required individual sessions, they get put into a group setting.’ (KI 2, female, SLT)

The PHC facilities and referring hospitals have built good working relationships, and they also hold cluster meetings to enhance better communication and a more effective referral process:

‘… [*P*]atients are being referred well … the referral routes are good … the communication has improved so much and has improved the referral system so much.’ (KI 6, male, PT)

On the basis of common responses to community needs, the rehabilitation professionals have formed relationships with other hospitals to develop standard operating procedures within various working groups to enhance interdisciplinary work efforts for integrated service delivery:

‘… [*C*]ollaborating with other hospitals … we … tried to formulate our own SOP [*standard operating procedures*] … within different task or work groups …’ (KI 6, male, PT)

## Discussion

Based on the articulated reality experienced by rehabilitation service providers, this study investigated perceptions regarding rehabilitation service integration at the PHC level.

### Rehabilitation services are not what they should be

Rehabilitation professionals at the coalface in the Johannesburg Metropolitan District in South Africa perceived the rehabilitation services as suboptimal and underdeveloped.^2^ Services lack comprehensiveness and fail to align with macro level health and PHC policies and guidelines. Policies and guidelines speak to the principles of access, equity and availability of comprehensive integrated rehabilitation services,^40,41,42^ which are not evident in the rehabilitation services in the Johannesburg Metropolitan District. Currently, the services are organised based on vertical programmes that focus on specific patient diagnoses, resulting in fragmented physical and mental health services.^10,19^ This lack of integration hinders effective service delivery and collaboration.^12^ Additionally, there is a disconnect between hospital referrals and the available resources in PHC, affecting the integration of rehabilitation services. The disparity in service availability, including human resources, greatly affects the continuum of care for service users.^30^ Notably, while physical rehabilitation is relatively advanced, mental health rehabilitation is severely lacking. The fragmentation of mental health and physical rehabilitation is evident not only at an operational level in terms of clinical management but also in the siloed approach to management, which indicates poor policy implementation of comprehensive rehabilitation services. A systematic review by Wakida et al. confirms the poor integration of rehabilitation services, particularly mental health, in PHC is a common occurance.^43^ The rehabilitation philosophy emphasises a comprehensive approach to patient care, through a clear and continuous person-centred approach.^43^ The process of being person centred implies the need to respond to patient needs and in this case mental health services are underrepresented.

In the current study, the clinic model prevailed in the CHCs in the Johannesburg Metropolitan District. According to McColl et al.,^22^ the clinic model is the most commonly observed PHC rehabilitation integration model in multi-provider PHC organisations. Despite bringing together various rehabilitation healthcare providers in one location, the clinic model, referred to as the mini-hospital structure by the participants, remains deeply ingrained in institutional-based medical practice patterns and culture. The clinic model primarily focusses on the acute phase of care for patients seen in CHCs, emphasising the intensive rehabilitation input and frequent intervention compared to the chronic phase, thus requires more time from rehabilitation professionals.^20^ Consequently, rehabilitation services are inadvertently confined within the CHC rather than out in the community,^44^ compromising health promotion and secondary preventive services as curative, institution-based medical model interventions take precedence.^20,44^ A skewed focus on curative services may reflect the burden of disease in South Africa. Within the clinic model, health professionals tend to deliver rehabilitation services within their usual scope of practice, often in a condition-specific and isolated manner. Such an approach has the potential to perpetuate siloed service provision, hindering the integration of rehabilitation in PHC.^22^ In a siloed approach inefficiency may manifest as patients are managed individually based on their diagnosis, resulting in lengthy waiting lists and treatment delays. Financial and geographical barriers may exacerbate healthcare inaccessibility.^15,45^

Unless specific measures are taken, locating services in the clinic setting may negatively affect the philosophy of integrated service delivery. An integrated service promotes the use of trans-, inter- and multidisciplinary approaches, which involves various interconnected health, social and environmental organisations to address patients’ and communities’ health care needs holistically.^25^ In a clinic setting, the patient as shown in this study is seen by individual therapists and data are captured separately with no clear system to integrate patient outcomes and interventions. Another criticism of the clinic model is that it removes patients from their social context, similar to institutional-based inpatient services.^22^ The services are often located far from patients’ homes and have long waiting times, leading to patients spending the entire day at the clinic waiting to be seen.^12^

It is necessary to select models that are tailored to specific services.^20^ Modifying or combining PHC rehabilitation integration models to foster interprofessional collaboration and effectively meet the needs of service users during the rehabilitation process can greatly enhance outcomes and enable rehabilitation professionals to adopt a holistic approach. One potential adaptation for integrated services is the outreach model, through task shifting and sharing to community rehabilitation workers, which offers notable advantages. Task shifting and task sharing in community-based teams were previously effectively applied in mental health services in India.^46^ The task shifting and task sharing model brings services closer to individuals, resulting in improved health outcomes, consistent care provision and reduced hospital admissions.^22^ The outreach model as mainly applied through rehabilitation professionals going out from the clinics into the community, while having its benefits, does not address the challenges faced by rehabilitation personnel in terms of costs, travel and accessibility in South Africa. Despite these obstacles, the participants demonstrated pragmatism and outlined measures to ensure the continuity of services.

### Despite the challenges, service delivery continues

Rehabilitation professionals in the Johannesburg Metropolitan District acknowledge the challenges they face in delivering effective services because of limited resources and contextual demands. Despite these obstacles, they remain optimistic and committed to service delivery, employing various strategies to mitigate these difficulties. Research suggests that health system actors, including rehabilitation personnel, utilise absorptive, adaptive and transformative strategies in response to the challenges they encounter.^47^

In the Johannesburg Metropolitan District, rehabilitation professionals have adapted their outreach strategies to suit their context. Although they face obstacles such as the cost and availability of transport,^44^ they collaborate with other sectors, such as the Department of Social Development, to conduct outreach campaigns and take advantage of the economies of scale. Intersectoral collaborations allow for the best possible use of resources and integration of public health rehabilitation models. In this case, they use intersectoral collaboration as outlined in the Community-based rehabilitation (CBR) model to rehabilitation integration.^48^ Intersectoral collaboration requires integrative cooperation of different health professionals and blending complementary competences and skills,^49^ which lead to service integration beyond the health sector.^22^ While the rehabilitation professionals may not have explicitly reviewed the components of CBR for a holistic approach, their commitment to intersectoral collaboration is a step in the right direction for adapting a PHC rehabilitation integration model and improving inter-organisational relationships and service delivery.^12,49^

Another mitigation strategy against limited resources and competing contextual demands used by rehabilitation professionals in this study is health promotion and prevention. The rehabilitation professionals interpreted the move to health promotion as part of the process of progressing patients from individual to group therapy and from health provider to patient focussed self-management and empowerment as seen in the use of peer support groups. The use of self-management as a model for integrated rehabilitation into PHC is effective because it embodies health promotion in which service users are the masters and drivers of their own healthcare.^22^ Self-management imparts new skills and information to patients, making them more confident and self-efficacious.^22^ The self-management model is compatible with the rehabilitation philosophy as it optimises independence, productivity and health-promoting social and economic life choices. Self-management alleviates the demand on service delivery because of high patient volumes, strengthens transdisciplinary working and consequently rehabilitation integration in PHC.^22^

The cooperation between the PHC, secondary and tertiary level hospitals is a commendable step towards integrated rehabilitation services. A functioning referral system connects clients and their families to community resources to enable effective rehabilitation.^50^ Promoting awareness of the rehabilitation service, expanding the services and building relationships with other professionals are additional adaptation strategies employed by rehabilitation professionals in this study. Examples of this include collaborations with other hospitals and formulation of standard operation procedural procedures within working groups. Valentijn et al.^12^ show that professional integration creates shared responsibility for service commissioning and fosters shared accountability, problem solving and decision-making to achieve optimal health and well-being.

One of the barriers to integrated service delivery is the lack of definition, awareness and recognition of the role of each professional within a multidisciplinary team. In support, Supper et al.^49^ indicate that the roles of each professional in collaborative relationships are often loosely defined, relying on trust and professional integration in the team. Indeed, there is common professional knowledge used by all healthcare professionals, and this knowledge is important for developing shared frames of reference and service delivery outcomes. Ultimately, integrated rehabilitation services are based on conceptual models that recognise that each profession has their own role to play in the rehabilitation process.^20^ Therefore, the concerted efforts by the rehabilitation professionals in the Johannesburg Metropolitan District to achieve integrated service delivery through awareness and professional relationship building must be supported and sustained by providing the necessary resources including adequate staffing. Awareness building and relationship building initiatives are fundamental to fostering interprofessional trust, health workforce efficiency^51^ and meso-level professional and organisational integration.^12^ Members of the healthcare team are more inclined to include rehabilitation in service delivery if they understand the services and outcomes that can be achieved.^52^

### Recommendations

The influx of acute patients in PHC facilities is influenced by the push for shorter hospital stays. Therefore, it is crucial to advocate for measures that strengthen the community level as a catchment area. Short-term solutions, such as adapted outreach campaigns, used to cope with the demands of treating patients in the acute phase of care must give way to more concrete and viable service delivery models and techniques. The outreach model through task shifting of rehabilitation service provision to community health workers, combined with the self-management model through community empowerment and peer support, needs to be explored and implemented through a well-informed strategy. The strategy should include adequate training and appropriate utilisation of community health workers.

Intersectoral collaboration, as indicated in the CBR model, between various governmental departments, along with advocacy for rehabilitation posts in other governmental departments, is needed. Furthermore, the shortage of rehabilitation personnel posts in PHC can be mitigated through the public-private partnerships proposed by the National Health Insurance (NHI). For example, 75% of occupational therapists work in the private sector, and similar figures may apply to other rehabilitation services. This presents an opportunity for rehabilitation professionals in private practice to be contracted into the public sector. The intersectoral nature of integrated service delivery and PHC requires a comprehensive multi-pronged approach that is contextually relevant and applicable across multiple settings. Existing rehabilitation service delivery models, such as the clinic model, need to be critically analysed and new models, which may involve a combination of the clinic model, outreach, self-management, case management, shared care and CBR, should be developed.

## Conclusion

The expressed reality is that rehabilitation services are not what they should be in the Johannesburg Metropolitan District and remain underdeveloped and poorly integrated into PHC, with suboptimal service provision and rooted in the medical model. We need to examine the factors that contribute to the difference in development and availability between physical and mental health services. Generalists at the PHC level are usually junior staff members who are overwhelmed with their responsibilities. They need to have access to real-time expertise, as is provided in a shared care model of service delivery, where they would be paired with more experienced personnel within the PHC team to promote collaboration that is more horizontal.
